# Triple-Band Notched Ultra-Wideband Microstrip MIMO Antenna with Bluetooth Band

**DOI:** 10.3390/s23094475

**Published:** 2023-05-04

**Authors:** Mohamed S. El-Gendy, Mohamed Mamdouh M. Ali, Ernesto Bautista Thompson, Imran Ashraf

**Affiliations:** 1 Microstrip Department, Electronics Research Institute, Cairo 11843, Egypt; mohamadalgendy2004@gmail.com; 2 Electrical Engineering Department, Faculty of Engineering, Assiut University, Assiut 71516, Egypt; mohamed.ali@ieee.org; 3 Higher Polytechnic School, Universidad Europea del Atlántico, Isabel Toress 21, 39011 Santander, Spain; ernesto.bautista@unini.edu.mx; 4 Department of Project Management, Universidad Internacional Iberoamericana, Campeche 24560, Mexico; 5 Project Management, Universidad Internacional Iberoamericana, Arecibo, PR 00613, USA; 6 Department of Information and Communication Engineering, Yeungnam University, Gyeongsan 38541, Republic of Korea

**Keywords:** ultra-wideband communication, microstrip antenna, sharpness edges, MIMO

## Abstract

In this paper, a novel ultra-wideband UWB antenna element with triple-band notches is proposed. The proposed UWB radiator element operates from 2.03 GHz up to 15.04 GHz with triple rejected bands at the WiMAX band (3.28–3.8 GHz), WLAN band (5.05–5.9 GHz), and X-band (7.78–8.51 GHz). In addition, the radiator supports the Bluetooth band (2.4–2.483 GHz). Three different techniques were utilized to obtain the triple-band notches. An alpha-shaped coupled line with a stub-loaded resonator (SLR) band stop filter was inserted along the main feeding line before the radiator to obtain a WiMAX band notch characteristic. Two identical U-shaped slots were etched on the proposed UWB radiator to achieve WLAN band notch characteristics with a very high degree of selectivity. Two identical metallic frames of an octagon-shaped electromagnetic band gap structure (EBG) were placed along the main feeding line to achieve the notch characteristic with X-band satellite communication with high sharpness edges. A novel UWB multiple-input multiple-output (MIMO) radiator is proposed. The proposed UWB-MIMO radiator was fabricated on FR-4 substrate material and measured. The isolation between every two adjacent ports was below −20 dB over the FCC-UWB spectrum and the Bluetooth band for the four MIMO antennas. The envelope correlation coefficient (ECC) between the proposed antennas in MIMO does not exceed 0.05. The diversity gains (DG) for all the radiators are greater than 9.98 dB.

## 1. Introduction

The communication link depending on ultra-wideband technology is a promising solution for numerous wireless networks, such as wireless body area networks (WBANs), Internet of Things (IoT) network systems, personal area networks (PAN), and remote sensing networks. The ultra-wideband communication network was selected because it uses a very high bandwidth radiator, which enables high-speed data transmission with low power consumption (i.e., −44.2 dBm) [1]. The UWB technologies utilize a very small pulse width (i.e., very large frequency range) through the transmission process over a short range distance (i.e., few meters) in smart homes, hospitals, etc. Therefore, the UWB radiator is an essential component of ultra-wideband network systems. In 2002, the US Federal Communications Commission (US-FCC) issued an unlicensed spectrum of bands ranging from 3.1 to 10.6 GHz with an operating bandwidth of 7.5 GHz for commercial applications. The profile of the ultra-wideband antenna design can be classified into a 3D-UWB antenna and a 2D-UWB antenna according to the requirements of the communication system [1,2]. The 3D-UWB antenna includes the biconical antenna, the discone antenna, and the log periodic dipole antenna. The 2D-UWB antenna includes planar antennas, printed/microstrip antennas, and dielectric resonator (DRA) antennas. The UWB microstrip radiators have numerous benefits, such as a small circuit size, low cost of fabrication, and the ability to connect with a different circuit. In [2], a printed ultra-wideband antenna fed by the coplanar waveguide (CPW) technique was reported. The reported antenna was designed and fabricated on FR-4 substrate material with relative permittivity of 4.4 and a substrate height of 1.6 mm. Both the radiating element with its feeding line and the ground plane were placed on the top layer of the substrate material. The radiating patch antenna was embedded within the rectangular slot of the ground plane and connected to the CPW input feed line. Since the FCC-UWB spectrum signal is utilized within a small distance (i.e., office, home, or hospital), it faces the main problem of interfering with other narrow unlicensed band signals such as WiMAX 3.5 GHz (3.3–3.8 GHz), WLAN 5.8GHz (5.15–5.85 GHz), and the X-band satellite communication (7.9–8.4 GHz) spectrum. The presence of these notch bands affects the efficiency of the communication link using the FCC-UWB spectrum. The solution to this issue is to utilize UWB antennas with single-, dual-, or triple-band notches at these narrow unlicensed spectra [3,4,5]. In [3], a printed dongle antenna used for ultra-wideband networks with a single narrow band rejection (i.e., WLAN) was reported. The radiator element was designed based on the self-complementary technique. A patch antenna with a Von Koch fractal shape at its boundary was designed. A rejected WLAN band was achieved by etching a fractal slot on the patch radiator near the CPW feed line. The reported monopole antenna was fabricated on FR-4 substrate material. In [4], an annular-ring monopole-printed antenna was reported to achieve ultra-wideband characteristics. The radiating element consists of an annular-ring-shaped monopole antenna. A pair of split ring resonators (SRRs) is placed on a superstrate over the CPW feed line to obtain dual band notches. In [5], an UWB-printed circular monopole patch radiator with a triple notch band was reported. The notch bands were achieved by loading triple split ring resonators (SRRs) on the opposite side of the CPW feeding line. One SSR is used for each notch band (i.e., 3.5 GHz WiMAX, 5.5 GHz WLAN, and 7.9 GHz X-band for satellite communication link). The printed antenna and SSRs are designed and fabricated on FR4-Epoxy material.

Since the UWB antenna uses an RF signal with low power and in the presence of some obstacles causing multipath fading, the multiple-input multiple-output (MIMO) network was introduced as a promising solution to solve this issue. Not only does the MIMO network system suppress multipath fading, but it also improves the network channel capacity [6]. Therefore, implementing the UWB antenna with MIMO technology increases the data rate, besides improving the channel capacity of the wireless communication link. The idea of using the MIMO network system is based on using one of the available diversity techniques (i.e., polarization diversity or spatial diversity). Thus, the investigation of the UWB antenna via the MIMO technique is based upon placing and arranging several antennas in a planar shape with different orientations or with a certain separation distance to achieve high multiplying gains [7,8,9,10]. The polarization diversity technique is selected rather than the spatial diversity one to investigate the UWB antenna with the MIMO system because the former introduces a smaller radiator board size than the second one. However, the UWB antenna in the MIMO system suffers from high mutual coupling between the adjacent radiators in the UWB-MIMO system. This is because of the small separated distance between every two adjacent radiators. In order to overcome this problem, the isolation level between the adjacent radiators should be better than a certain level in dB to guarantee alleviation of the interference between the two coupling currents. In [11], the concept of the MIMO capacity and symbol error rate relies on an isolation level between −15 dB and −17 dB in order to overcome the mutual coupling between the radiators. Several techniques have been reported to improve the isolation between the adjacent ports in the UWB-MIMO system [12,13,14,15]. These techniques involve placing stubs between the radiators connected to the ground plane [12], placing neutralization lines [13], using electromagnetic bandgap structures (EBGs) [13], and using defective ground structures (DGSs) [15]. Defective ground structures (DGSs) are considered a promising method to improve the isolation between adjacent or different ports.

In this paper, four ports of an UWB-MIMO structure were fabricated on a low-cost FR-4 substrate with relative permittivity of $\varepsilon_{r}$ = 4.5, a loss tangent of 0.025, and a height of h=1.5 mm. The radiator element used in the proposed MIMO configuration operates from 2.03 GHz to 15.04 GHz with triple rejected bands at the WiMAX band (3.28–3.8 GHz), WLAN band (5.05–5.9 GHz), and X-band (7.78–8.51 GHz). In addition, it supports the Bluetooth band (2.4–2.483 GHz). The proposed radiator is available to operate in FCC-UWB networks. A coupled line with a stub-loaded resonator (SLR), etching two U-shaped slots on the radiator, and an octagon-shaped EBG structure are utilized to achieve the triple band notches at the WiMAX band, WLAN band, and X-band, respectively. The obtained notch bands have very high selectivity at the beginning and the end of the notch bands. The MIMO configuration circuit has one connected ground plane. High isolations were obtained between the four radiators by using the defective ground structure (DGS) technique.

The article is arranged as follows. The design of a UWB antenna element is explained in Section 2. In Section 3, the design of the UWB-MIMO antenna is introduced. The results and discussion are given in Section 4. Finally, Section 5 provides the conclusions.

## 2. Design of an Ultra-Wideband Antenna Element

**Figure 1:**
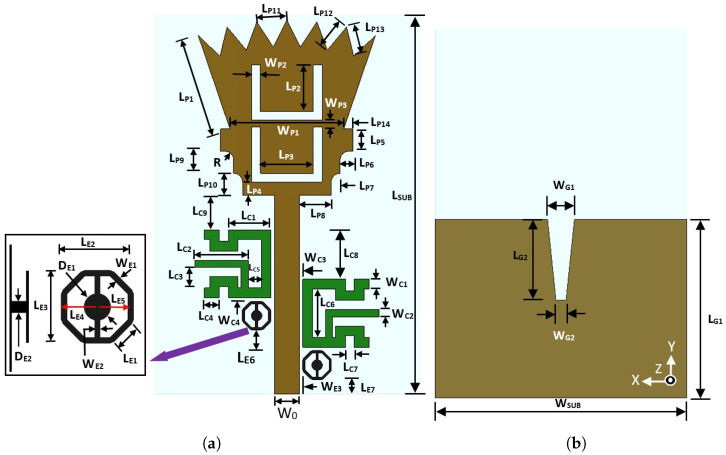
Proposed UWB microstrip monopole antenna. (**a**) Antenna layer (top view). (**b**) Ground plane layer (bottom view).

Figure 1 shows the geometry and parameters of the proposed UWB monopole microstrip antenna element. Table 1 shows the values for the proposed antenna design. The proposed UWB microstrip antenna was designed on FR-4 dielectric substrate material with relative permittivity of $\varepsilon_{r}$ = 4.5, a dielectric loss tangent of 0.025, and a substrate height of *h* = 1.5 mm. The proposed UWB antenna element consists of three sections placed on the top layer of the dielectric substrate. The first one is a 50 Ω microstrip feeding line with a width of 2.8 mm. This section represents the input port section and it should be electrically consistent with the input RF power source devices. The second section is a stepped isosceles trapezoidal section. It represents the interface between the 50 Ω microstrip feeding line and the patch radiator. It is used to match the input impedance of the main feeding line and the input impedance of the UWB patch radiator. The third section is the UWB microstrip radiator. A partially metallic ground plane layer with an etched trapezoidal slit is located at the bottom side of the dielectric material. The partial ground plane is selected rather than the full ground plane for obtaining ultra-wide impedance bandwidth. The trapezoidal slit that was etched on the top edge of the partial ground plane was selected to match the high input impedance of the monopole patch antenna and 50 Ω microstrip feeding line. The overall dimensions of the proposed antenna are 43 × 30 × 1.5 mm${}^{3}$.

### 2.1. Step 1: UWB Microstrip Monopole Antenna Element Design

**Figure 2:**
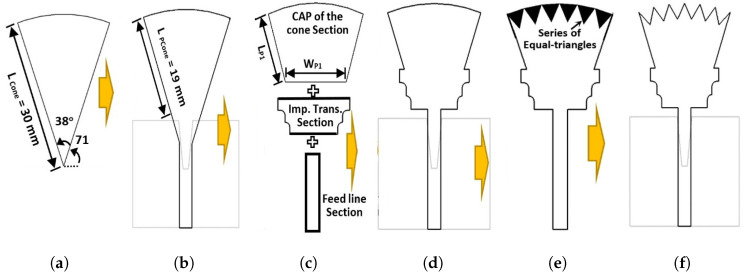
The UWB microstrip monopole antenna: design steps. (**a**) Planar cone; (**b**) Case 1; (**c**) Case 2; (**d**) Case 3; (**e**) Case 4; (**f**) Case 5.

The first step in designing an UWB microstrip monopole antenna is to select the radiator shape. Figure 2 shows the design steps in designing the proposed UWB antenna. The projection plane of the cone (i.e., planar cone shape) is chosen to be the radiator shape of the proposed UWB antenna, as shown in Figure 2a. The chosen planar cone shape has a side edge length of 30 mm and the internal angle between its two-sided edges is 38 degrees. Since the required frequency for the proposed antenna is the FCC-UWB spectrum that operates from 3.1 to 10.6 GHz, in addition to the Bluetooth band at a frequency of 2.4 GHz, the electrical length of the planar cone ($L_{Pcone}$) is calculated at the operating frequency of 2.4 GHz, which is approximately equal to a quarter wavelength, as shown in Equations (1)–(3).

(1) $$L_{Pcone}=\frac{\lambda_{g}}{4}$$

(2) $$\lambda_{g}=\frac{C}{f_{0}\sqrt{\epsilon_{eff}}}$$

(3) $$\epsilon_{eff}=\frac{\varepsilon_{r}+1}{2}+\frac{\varepsilon_{r}-1}{2}{\left[1+12\frac{h}{W_{0}}\right]}^{-0.5}$$

where $\lambda_{g}$ is the waveguide wavelength, *C* is the speed of light ($C=3\times 10^{8}$ m/s), $f_{0}$ is the operating frequency (i.e., $f_{0}=2.4$ GHz), $\varepsilon_{r}$ is the relative permittivity (i.e., $\varepsilon_{r}$ = 4.5), $\epsilon_{eff}$ is the effective relative permittivity, and $W_{0}$ is the 50-ohm feed line width.

**Figure 3:**
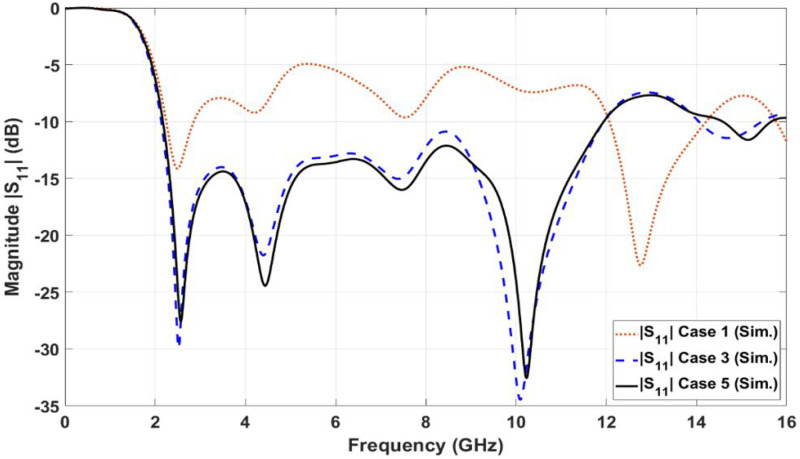
Simulated reflection coefficients ($|S_{11}|$) in dB for UWB antenna design steps.

Figure 2b shows the first case (case 1) in designing the UWB microstrip antenna. In case 1, the planar cone monopole antenna with a side edge length of 19 mm is connected to the 50-ohm microstrip feeding line. The microstrip cone antenna and its feeding line are located on the top layer of the dielectric substrate. A partial ground plane layer with an etched trapezoidal slit is located at the bottom side of the dielectric substrate material. The aim of the etched trapezoidal slit at the top edge of the partial ground plane is to match the high input impedance of the monopole patch antenna and 50 Ω microstrip feeding line. The design in Figure 2b was simulated using CST microwave studio. Figure 3 shows the reflection coefficient (|$S_{11}$| in dB) of the first case (for case 1) design. As shown in Figure 3, the reflection coefficient curve (case 1 curve) is not matched within the required operating bandwidth (i.e., FCC-UWB spectrum). The |$S_{11}$| curve for case 1 oscillates between −5 and −10 dB. The reason behind the mismatching is that the input impedance of the planar cone antenna is very high, while the impedance of the feeding line is 50 ohms.

According to the aforementioned mismatching characteristics, the next design in Figure 2c was introduced to overcome this issue. The modified design in Figure 2c consists of three connected sections at the top layer of the dielectric substrate: a cap for the planar cone section, an impedance transformer section, and the feeding line section. The cap of the cone section is the top part of the planar cone of Figure 2a with a side edge length of $L_{P1}$ = 11.19 mm and bottom width of $W_{P1}$ = 12.84 mm. The impedance transformer section is placed to match the high input impedance of the planar cone radiator and the 50-ohm feeding line. It is composed of a trapezoidal shape with a stepped leg. The long base of the trapezoidal shape is connected to the bottom edge of the planar cap of the cone radiator, while the small base of the trapezoidal shape is connected to the 50-ohm feeding line. As shown in Figure 3, the simulated reflection coefficient curve (for case 3) is matched within the required operating bandwidth from 2.16 to 11.96 GHz. In case 4, a series of six equal triangles is etched from the top edge of the cap of the cone radiator in order to improve the matching characteristics (i.e., reflection coefficient performance) at the frequency of 8.4 GHz. As shown in Figure 3, the simulated reflection coefficient curve (for case 5) is fully matched within the required operating bandwidth from 2.17 to 11.96 GHz. The simulated bandwidth for the UWB monopole microstrip antenna for case 5 is 9.79 GHz (BW% = 138.57%), which is sufficient to cover the FCC-UWB spectrum. Moreover, the antenna supports the Bluetooth frequency band that operates from 2.4 to 2.483 GHz.

### 2.2. Step 2: UWB Antenna with Single Band Notch at WiMAX Band

**Figure 4:**
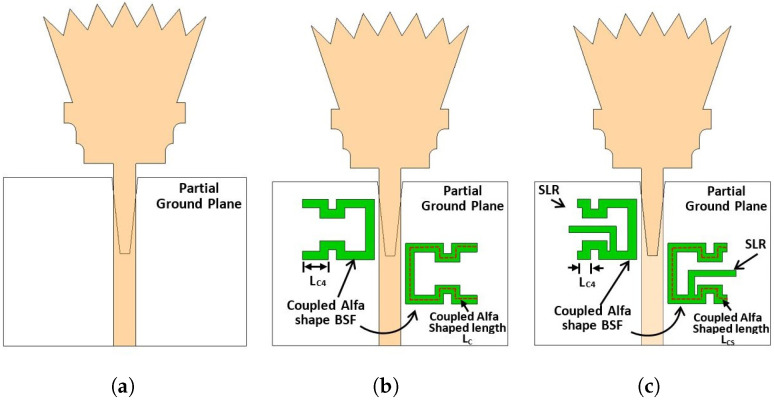
UWB antenna with single band notch: design steps. (**a**) Case 5; (**b**) Case 6; (**c**) Case 7.

The UWB spectrum signal faces some sort of interference with other narrow unlicensed band signals such as WiMAX since it operates at a small distance, e.g., an office, home, or hospital. Therefore, the WiMAX band extending from 3.3 to 3.8 GHz should be excluded from the obtained FCC-UWB signal. In order to achieve WiMAX band rejection over the aforementioned frequency range, the coupled line technique is introduced. An alpha-shaped coupled line band stop filter was placed along the feeding line before the proposed UWB radiator to obtain a band notch characteristic at the WiMAX frequency band, as shown in Figure 4b. The coupled line length for the alpha-shaped filter is calculated from Equation (4) at the notch frequency of $f_{notch}=3.3$ GHz. The value of the calculated coupled line length is $L_{C}=25.02$ mm (i.e., electrical length = 0.5 $\lambda_{g}$) while the simulated value is $L_{C}=28.92$ mm (i.e., electrical length = 0.558 $\lambda_{g}$).

(4) $$L_{coupled}≃\frac{\lambda_{g}}{2}=\frac{C}{2f_{notch}\sqrt{\epsilon_{eff}}}$$

where $\lambda_{g}$ is the waveguide wavelength, *C* is the speed of light (C= 3 $\times 10^{8}$ m/s), $f_{0}$ is the operating notch frequency at the WiMAX band, and $\epsilon_{eff}$ is the effective relative permittivity.

**Figure 5:**
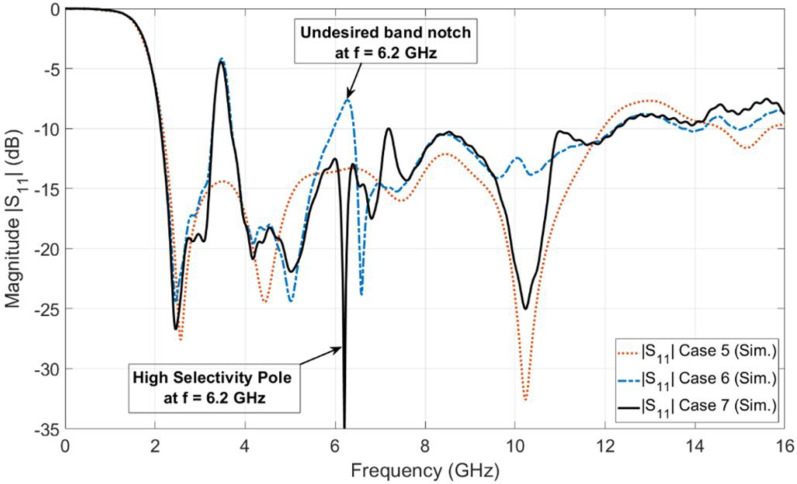
Simulated reflection coefficients for UWB antenna with WiMAX band notch.

**Figure 6:**
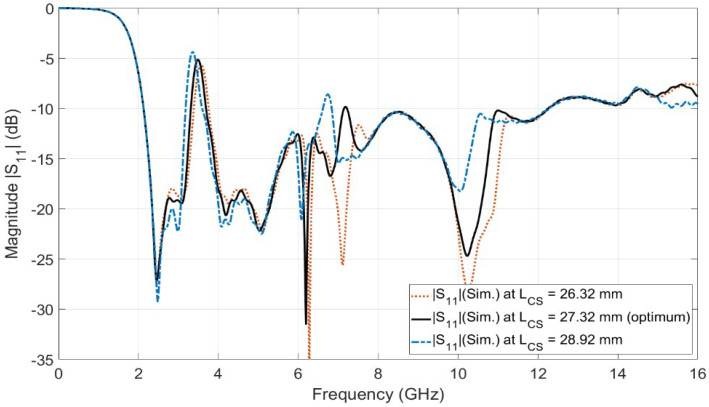
The parametric study of the length $L_{CS}$.

Figure 5 shows the simulated reflection coefficient for the design of Figure 4b (i.e., Case 6). It is shown from the figure that the antenna operates over the FCC-UWB spectrum with a WiMAX band notch that extends from 3.3 to 3.8 GHz. However, there is another undesired band notch (i.e., first-order mode) at the frequency of 6.2 GHz. In order to overcome the undesired band notch, a stub-loaded resonator (SLR) is added to the alpha-shaped coupled line BSF, as shown in Figure 4c. Not only does the stub-loaded resonator (SLR) introduce a high selectivity characteristic but it also minimizes the length of the half waveguide coupled line (i.e., alpha-shaped coupled line length). As shown in Figure 4b,c, the length of the coupled alpha shape is reduced from $L_{C}$ = 28.92 mm to $L_{CS}$ = 27.32 mm. Figure 6 shows the investigation of the influence of $L_{CS}$. Three values of $L_{CS}$ are selected, 26.32 mm, 27.32 mm, and 28.92 mm, while the other parameters are kept constant. It is clear from Figure 6 that the optimum curve is at $L_{CS}$ = 27.32 mm. The simulated reflection coefficient for the proposed UWB antenna with a WiMAX band notch using coupled line and SLR (i.e., case 7) is presented in Figure 5. It is depicted by the case 7 curve in Figure 5 that the proposed antenna operates from 2.12 to 12.24 GHz with a band notch from 3.3 to 3.8 GHz. Moreover, the antenna supports the Bluetooth frequency band operating from 2.4 to 2.483 GHz.

### 2.3. Step 3: UWB Antenna with Dual-Band Notch at WiMAX Band and WLAN Band

**Figure 7:**
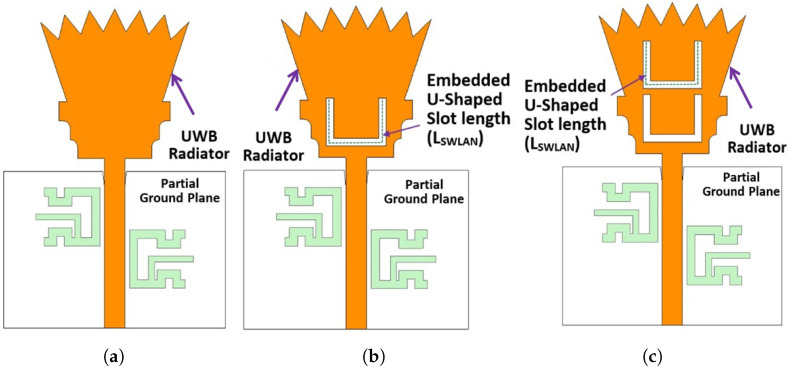
UWB antenna with dual-band notches: design steps. (**a**) Case 7; (**b**) Case 8; (**c**) Case 9.

The third step is to place a second band notch at the WLAN operating frequency from 5.15 to 5.85 GHz at the UWB spectrum signal. The WLAN band notch is required to overcome the interference between the obtained FCC-UWB signal and the local unlicensed WLAN band signal. In order to achieve WLAN band rejection over the aforementioned frequency range, a single U-shaped slot is embedded and etched at the bottom side of the proposed UWB radiator, as shown in case 8 of Figure 7b. The length of the etched U-shaped slot is calculated from Equation (5) at the notch frequency of $f_{notch}$ = 5.5 GHz. The value of the calculated U-shaped slot length is $L_{SWLAN}$ = 14.87 mm (i.e., electrical length = 0.5 $\lambda_{g}$) while the simulated value is $L_{SWLAN}$ = 18.5 mm (i.e., electrical length = 0.62 $\lambda_{g}$).

(5) $$L_{Embedded}≃\frac{\lambda_{g}}{2}=\frac{C}{2f_{notch}\sqrt{\epsilon_{eff}}}$$

where $\lambda_{g}$ is the waveguide wavelength, *C* is the speed of light ($C=3\times 10^{8}$ m/s), $f_{0}$ is the operating notch frequency at the WLAN band, and $\epsilon_{eff}$ is the effective relative permittivity.

**Figure 8:**
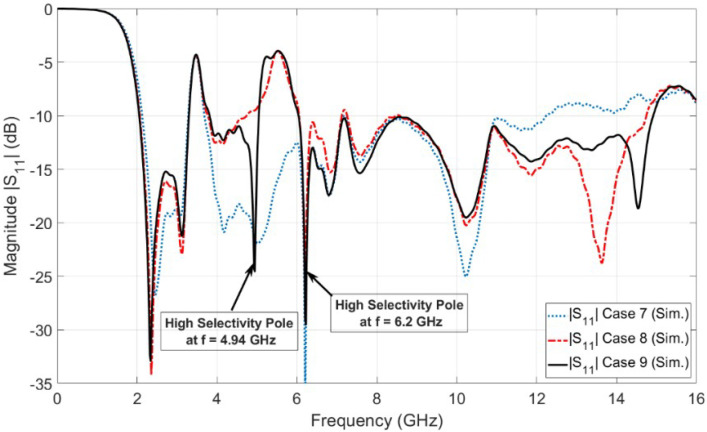
Simulation reflection coefficients for UWB antenna with dual-band notch.

**Figure 9:**
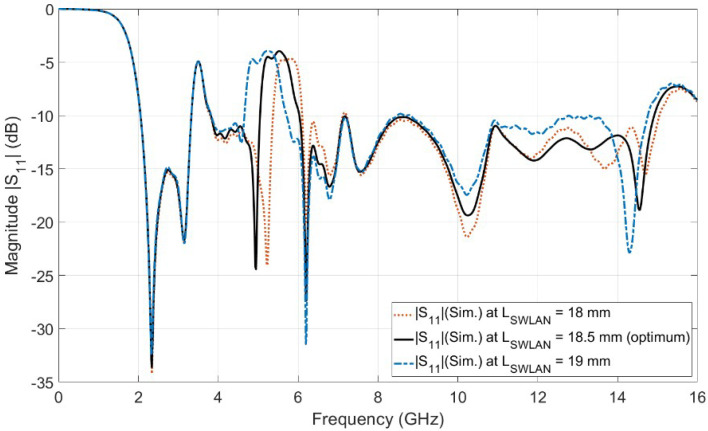
The parametric study of the length $L_{SWLAN}$.

Figure 8 shows the simulated reflection coefficient (|$S_{11}$| in dB) for case 8 (i.e., single etched U-shaped slot) in Figure 7b. It is observed from the simulated curve of Figure 8 (case 8) that the antenna operates from 2.06 to 14.74 GHz (i.e., UWB spectrum) with dual-band notches at WiMAX and WLAN. The WLAN band notch extended from 4.73 GHz to 6.05 GHz. Moreover, it is observed from Figure 8 (case 8) that the WLAN band notch has high selectivity (i.e., higher sharpness edge) at the end of the band, while it has low selectivity (i.e., lower sharpness edge) at the beginning of the band. Therefore, another U-shaped slot is etched on the top of the UWB radiator in order to improve the selectivity characteristic at the beginning of the WLAN band notch. The two U-shaped slots are vertically aligned and have the same length of $L_{SWLAN}$, as shown in Figure 7c. The vertically separated distance between the centers of the U-shaped slots is the equal quarter wavelength ($\lambda_{g}$/4) to obtain the second high selectivity at the beginning of the band. Placing two vertically aligned U-shaped slots with a separated distance of a quarter wavelength introduces a very high sharpness edge or high selectivity characteristic at the beginning of the WLAN band. The high selectivity occurs between the transmission band of the radiated UWB signal and the rejected one at the WLAN notch band, as shown in Figure 8. This high selectivity characteristic is required for protecting the receiver from damage or even preventing overlap between the radiating UWB RF signal and the indoor/local WLAN signal. Figure 9 shows the investigation of the effect of $L_{SWLAN}$ in both slots. Three values of $L_{SWLAN}$ are selected, 18 mm, 18.5 mm, and 19 mm, while the other parameters are kept constant. It is clear from Figure 9 that the optimum curve is at $L_{SWLAN}$ = 18.5 mm. Figure 8 (case 9) depicts the simulated reflection coefficient (|$S_{11}$| in dB) of the UWB antenna with dual-band notches at the WiMAX band and WLAN band. The UWB antenna with dual-band notches operates from 2.05 to 14.92 GHz, while the the WiMAX band and the WLAN band extend from 3.29 to 3.81 GHz and from 5.05 to 6.02 GHz, respectively. It is shown from Figure 8 (case 9) that the antenna supports the FCC-UWB signal and rejects the indoor WiMAX and WLAN narrow-band RF signals. In addition to this, the antenna supports the Bluetooth band that operates from 2.4 to 2.483 GHz. Moreover, it is observed that there is a very high sharpness edge or high selectivity characteristic at the beginning of the WLAN band at a frequency of *f* = 4.94 GHz. Therefore, the WLAN band notch is located between two very high sharpness edges (high selectivity poles), one at the beginning of the WLAN band at a frequency of *f* = 4.94 GHz and the other one located at the end of the band at a frequency of *f* = 6.2 GHz. Thus, the WLAN band notch resembles a rectangular shape. This rectangular notch band improves the radiating characteristic of the antenna more than the conventional notch antenna (i.e., single U-shaped slot UWB radiator).

### 2.4. Step 4: UWB Antenna with Triple Notch at WiMAX Band, WLAN Band, and X-Band (Proposed)

**Figure 10:**
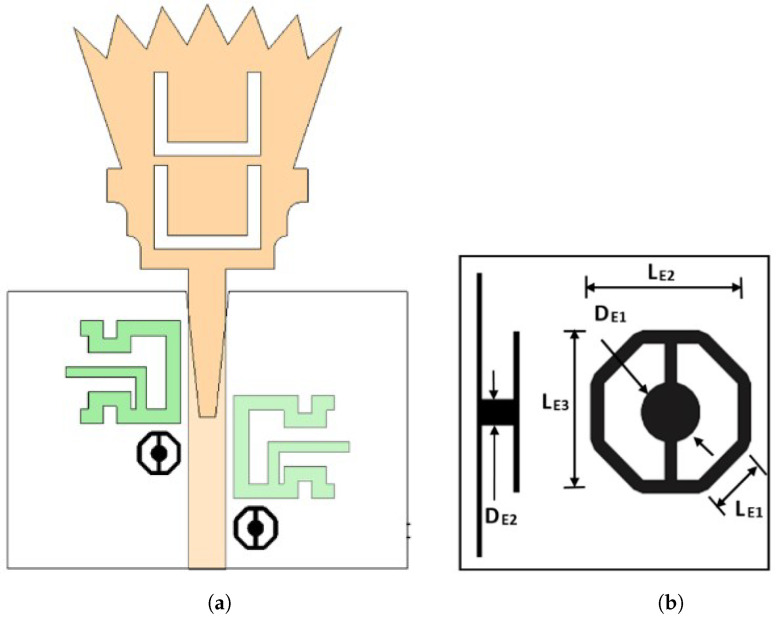
Proposed UWB antenna with triple band notches. (**a**) Case 10; (**b**) EBG unit cell.

**Figure 11:**
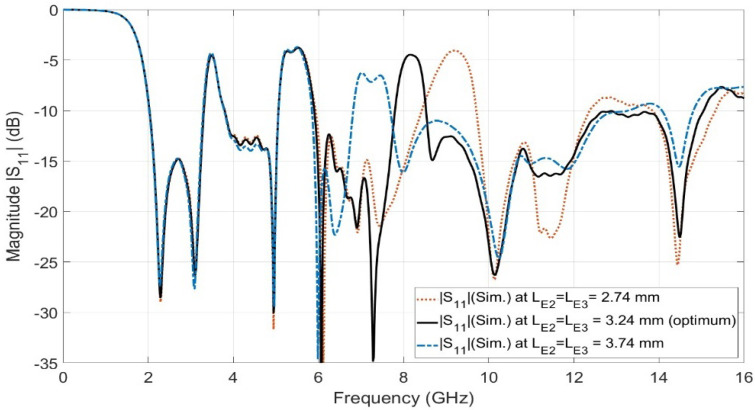
The parametric study of the lengths $L_{E2}$ and $L_{E3}$.

**Figure 12:**
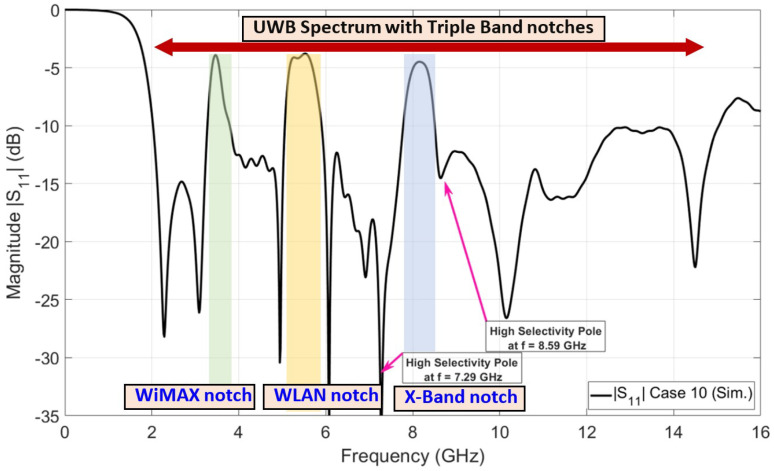
Reflection coefficient of proposed UWB antenna with triple band notches.

The final step in designing the proposed UWB antenna element with a triple band notch is to design a third band notch for X-band satellite communication that operates from 7.9 to 8.4 GHz. The X-band satellite communication band notch is required to be taken into account to overcome the interference between the obtained FCC-UWB signal and the X-band satellite signal. Electromagnetic band gap structure (EBG) technology is introduced to design a band stop filter to reject the licensed X-band satellite communication band over the aforementioned frequency range. A frame composed of an octagon-shaped EBG was proposed to achieve a band notch characteristic at X-band satellite communication in the UWB antenna, as shown in Figure 10, right view. Two identical frames of octagon-shaped EBG are placed along the 50-ohm feeding line with a vertically separated distance of a quarter wavelength ($\lambda_{g}$/4). The frame of the octagon-shaped EBG is connected to the metallic via (i.e., shorting pin) by using two vertically microstrip ties. The metallic via connects the octagon-shaped EBG to the ground plane. Placing two EBG unit cells with a separated distance of a quarter wavelength introduces a high sharpness edge at both the beginning and the end of the X-band satellite communication notch band. The horizontal length ($I_{E2}$) and the vertical length ($I_{E3}$) of the EBG unit represent important parameters in adjusting the required stop-band characteristics. Figure 11 shows the investigation of the effect of $L_{E2}$ and $L_{E3}$. The two lengths $L_{E2}$ and $L_{E3}$ are equal in value. Three values of $L_{E2}$ and $L_{E3}$ are selected, 2.74 mm, 3.24 mm, and 3.74 mm, while the other parameters are kept constant. It is clear from Figure 11 that the optimum curve is at $L_{E2}=L_{E3}$ = 3.24 mm. Figure 12 shows the simulated reflection coefficient (|$S_{11}$| in dB) of the proposed UWB antenna with triple band notches at the WiMAX band, WLAN band, and X-band. It is shown in Figure 12 that the proposed UWB antenna operates from 2.03 GHz up to 15.04 GHz. The proposed antenna rejects triple bands, namely the WiMAX band (from 3.28 GHz to 3.8 GHz), WLAN band (from 5.05 GHz to 5.9 GHz), and X-band (from 7.78 GHz to 8.51 GHz). The proposed antenna supports the Bluetooth frequency that operates from 2.4 to 2.483 GHz. Moreover, it is observed that the X-band notch is located between two sharp edges. One is located at the beginning of the X-band notch and the other is located at the end of the X-band at frequencies of *f* = 7.29 GHz and *f* = 8.59 GHz, respectively. Adding the Bluetooth band to the UWB signal is very useful for establishing a communication channel between unlicensed bands for users/patients and diagnostic devices in biomedical applications.

## 3. Design of Ultra-Wideband MIMO Antenna

**Figure 13:**
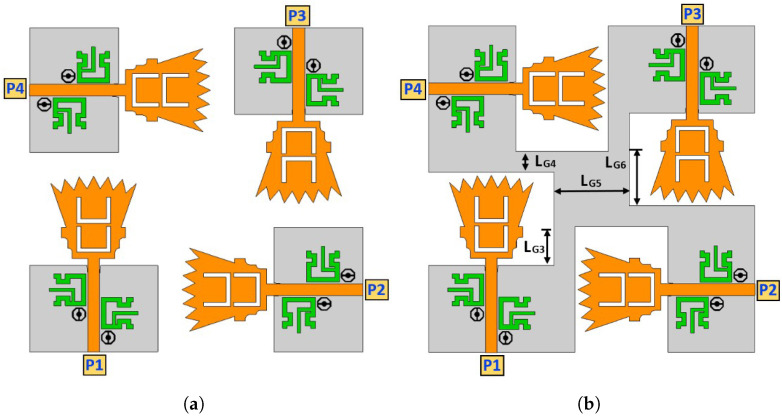
UWB-MIMO antenna design: ground plane study. (**a**) Non-connected ground plane; (**b**) connected ground plane.

**Figure 14:**
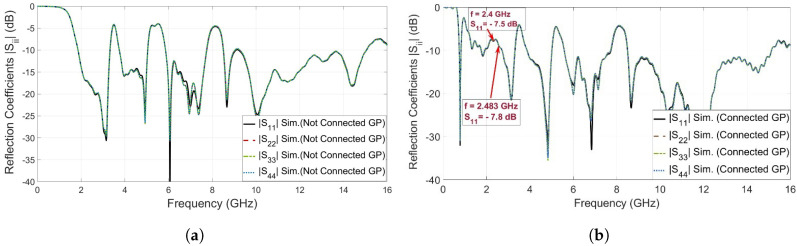
Reflection coefficients for the UWB-MIMO antenna. (**a**) Non-connected ground plane; (**b**) connected ground plane.

Four proposed UWB antenna elements are perpendicularly placed to each other in order to design a MIMO structure, as shown in Figure 13a. In Figure 13a, the four UWB-MIMO antennas are placed with separate ground planes (i.e., non-connected ground planes), where the port isolation characteristics seem very high [16,17]. The high isolation characteristics for the separated ground planes appear since there is no common coupling current passing through the ground panes (GPs). In practical systems, MIMO antennas with a separated ground plane topology are not preferable since the signal levels at all ground planes (i.e., zero volts) are not the same. UWB-MIMO antennas with a common ground plane configuration (i.e., connected ground planes) are utilized in order to increase the system reliability, as shown in Figure 13b. Here, a square metallic connection is tied to the four ground planes by four metallic strips. Table 2 shows the values of the parameters for the ground plane connection. Both UWB-MIMO antennas with the separate ground plane (i.e., non-connected GP) configuration and UWB-MIMO antennas with the common ground plane (i.e., connected GP) configuration are simulated using the CST microwave studio simulator. Figure 14a shows the four reflection coefficients (i.e., |$S_{ii}$| in dB, *i* is 1, 2, 3, and 4) at ports 1, 2, 3, and 4 of the UWB-MIMO antenna with the separate ground plane (i.e., non-connected GP) configuration. It is shown that the FCC-UWB spectrum with triple band notches was obtained in addition to the Bluetooth band. On the other hand, Figure 14b shows the four reflection coefficients (i.e., |$S_{ii}$| in dB, *i* = 1, 2, 3, and 4) at ports 1, 2, 3, and 4 of the UWB-MIMO antenna with the common ground plane (i.e., connected GP) configuration. It is noticed that the FCC-UWB spectrum with triple band notches was obtained but there is a mismatching characteristic at the Bluetooth band due to the coupling between the UWB antenna elements.

**Figure 15:**
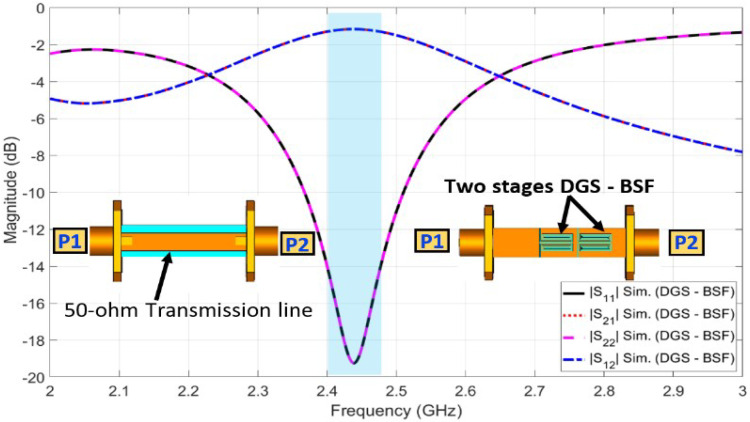
Reflection coefficient and transmission coefficient for BSF.

**Figure 16:**
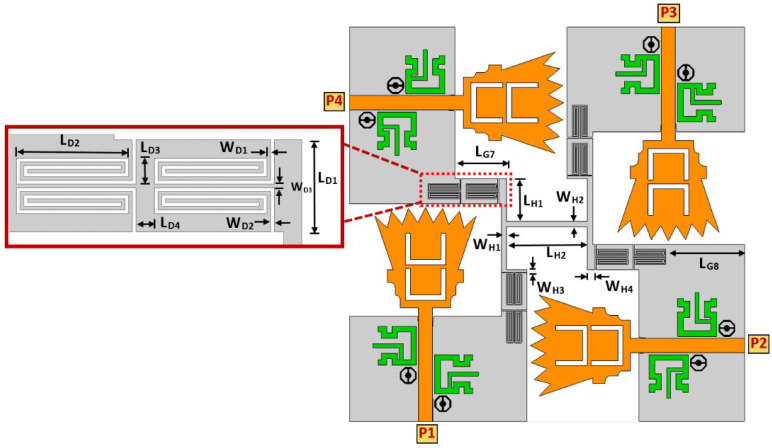
Proposed UWB-MIMO antenna design with DGS.

**Figure 17:**
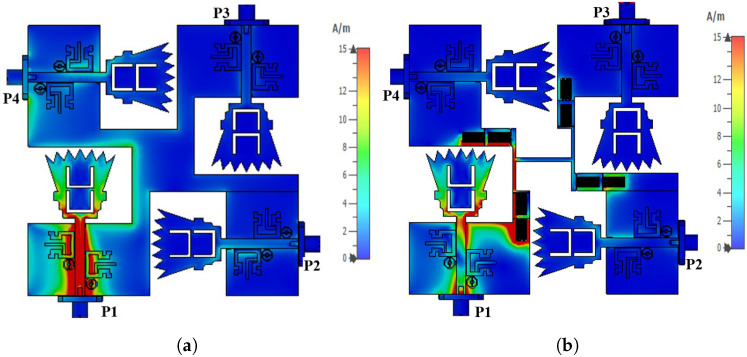
The surface current distributions for UWB-MIMO antenna at f = 2.44 GHz. (**a**) UWB-MIMO antenna without DGS; (**b**) UWB-MIMO antenna with DGS.

In order to overcome the problem mentioned above, a defective ground structure (DGS) band stop filter (BSF) was proposed and designed at the Bluetooth band (i.e., 2.4–2.483 GHz). The two-port DGS-BSF was simulated in CST microwave studio, as shown in Figure 15. The DGS-BSF consists of two stages of two defected coiled slits on the ground plane underneath a 50-ohm microstrip transmission line. The length of each slit is 27.6 mm (0.41 $\lambda_{g}$) while the width of the slit is 0.2 mm. Figure 15 shows the reflection coefficients (i.e., |$S_{11}$| and |$S_{22}$| in dB) and the transmission coefficients (i.e., |$S_{21}$| and |$S_{12}$| in dB) for the DGS band stop filter (BSF). It is shown from the figure that there is a band-stop characteristic over the Bluetooth operating frequency (2.4–2.483 GHz). Four DGS-BSFs were placed and embedded between the four ground planes of four UWB antennas and connected together by an H-shaped strip ground plane, as shown in Figure 16. Table 2 shows the values of the parameters for the ground plane connection and BSF. In order to demonstrate the effect of the four DGS–BSF, the surface current distributions for both the UWB-MIMO antenna without DGS and the UWB-MIMO antenna with DGS at *f* = 2.44 GHz are shown in Figure 17. In Figure 17a, as the first antenna is excited, the surface current is highly induced to the fourth antenna via the metallic connection between them. This induction causes a mismatch characteristic at the Bluetooth band. Meanwhile, in Figure 17b, as the first antenna is excited, there is no surface current induced by any antenna elements in MIMO thanks to the fourth DGS-BSF between them.

## 4. Results and Discussion

**Figure 18:**
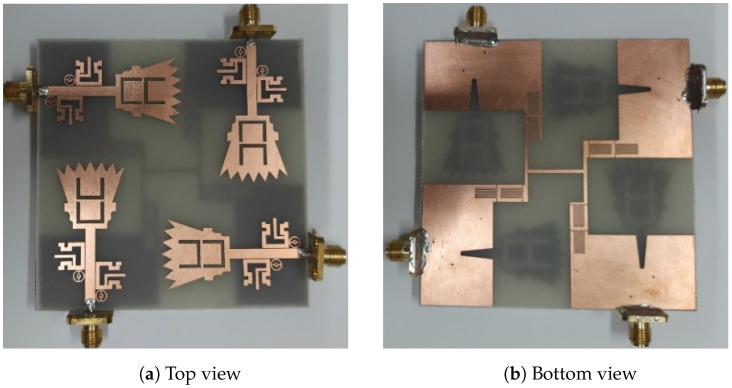
Photographs of the proposed UWB-MIMO antenna.

The proposed UWB-MIMO antenna was fabricated on FR-4 material with relative permittivity of $\varepsilon_{r}$ = 4.5, a loss tangent of 0.025, and a substrate height of *h* = 1.5 mm. Figure 18 shows the fabricated UWB-MIMO antennas. The overall size of the proposed UWB-MIMO antenna is 78 × 78 × 1.5 mm${}^{3}$.

### 4.1. S-Parameter Characteristics for UWB-MIMO Antennas

**Figure 19:**
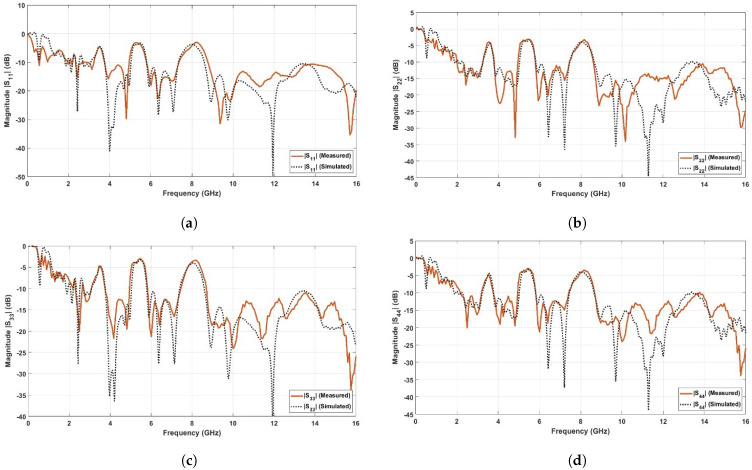
Measured and simulated reflection coefficients for proposed UWB-MIMO antenna. (**a**) |$S_{11}$| in dB at port (1); (**b**) |$S_{22}$| in dB at port (2); (**c**) |$S_{33}$| in dB at port (3); (**d**) |$S_{44}$| in dB at port (4).

**Figure 20:**
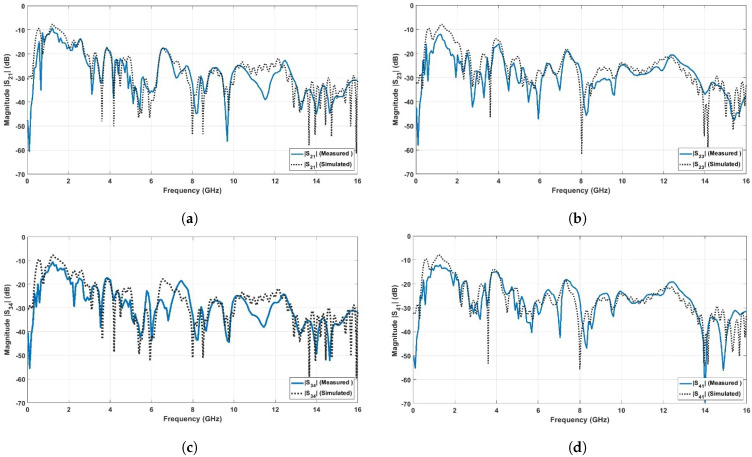
Measured and simulated isolation for proposed UWB-MIMO antenna. (**a**) |$S_{21}$| in dB between port (1) and port (2); (**b**) |$S_{23}$| in dB between port (2) and port (3); (**c**) |$S_{34}$| in dB between port (3) and port (4); (**d**) |$S_{41}$| in dB between port (4) and port (1).

Figure 19 shows the simulated and the measured reflection coefficients (|$S_{11}$|, |$S_{22}$|, |$S_{33}$|, and |$S_{44}$| in dB) of the proposed UWB-MIMO antennas. The experimental curves were obtained by using the Rohde & Schwarz VNA Model of ZVA67, Rohde & Schwarz, Columbia, MD, USA. It is depicted that the four MIMO antennas operate in the FCC-UWB spectrum with triple band notches at the WiMAX band (3.3–3.8 GHz), WLAN band (5.15–5.85 GHz), and X-band (7.9–8.4 GHz). Moreover, the proposed MIMO antennas support the Bluetooth band that extends from 2.4 to 2.483 GHz. Figure 20 shows the simulated and the measured port isolation (|$S_{21}$|, |$S_{23}$|, |$S_{34}$|, and |$S_{41}$| in dB) between the proposed UWB-MIMO antennas. It is observed that the curves of the isolation levels are below −20 dB over the FCC-UWB and the Bluetooth band for the four MIMO antennas.

### 4.2. Radiation Pattern and Realized Gain Characteristics for UWB-MIMO Antennas

**Figure 21:**
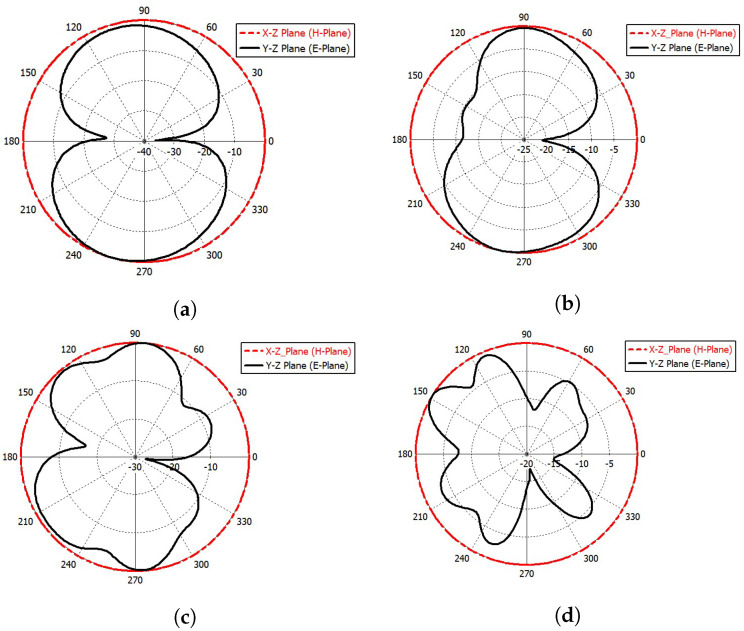
The simulated far-field radiation pattern at port (1). (**a**) E-plane and H-plane at *f* = 2.4 GHz; (**b**) E-plane and H-plane at *f* = 4 GHz; (**c**) E-plane and H-plane at *f* = 6.6 GHz; (**d**) E-plane and H-plane at *f* = 10 GHz.

**Figure 22:**
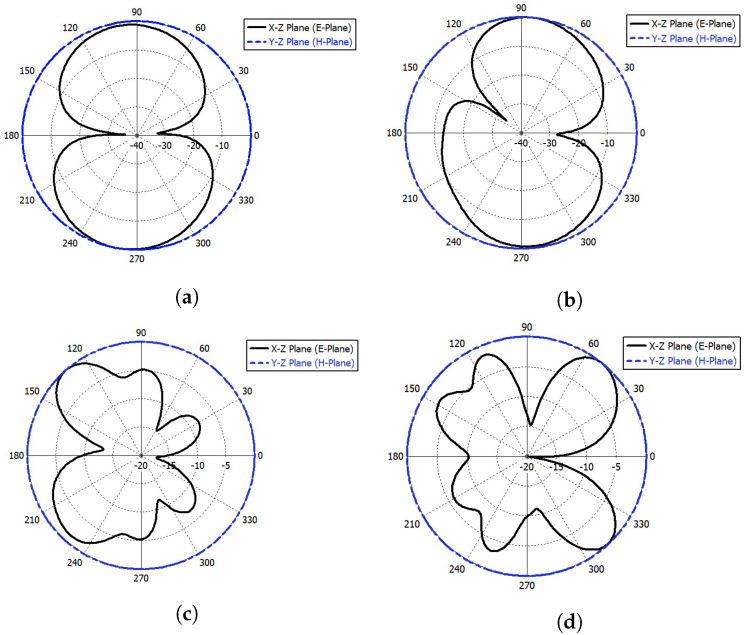
The simulated far-field radiation pattern at port (4). (**a**) E-plane and H-plane at *f* = 2.4 GHz; (**b**) E-plane and H-plane at *f* = 4 GHz; (**c**) E-plane and H-plane at *f* = 6.6 GHz; (**d**) E-plane and H-plane at *f* = 10 GHz.

**Figure 23:**
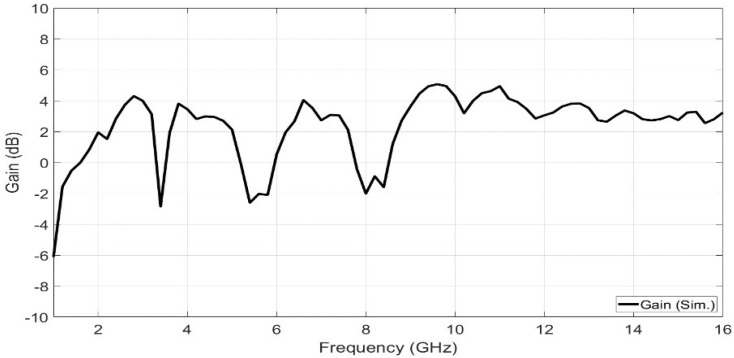
The simulated gain versus the operating frequency range.

Figure 21 and Figure 22 show the simulated far-field radiation patterns for port (1) and port (4) at frequencies of 2.4 GHz, 4 GHz, 6.6 GHz, and 10 GHz, respectively. Figure 23 shows the simulated gain curve versus the range of the desired spectrum.

### 4.3. Diversity Analysis Characteristics (ECC, DG, and TARC) for Proposed UWB-MIMO Antennas

**Figure 24:**
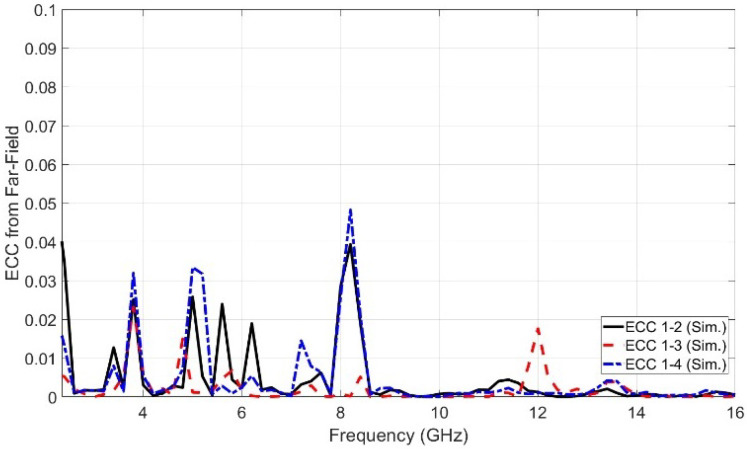
The simulated ECC versus frequency generated from far field.

**Figure 25:**
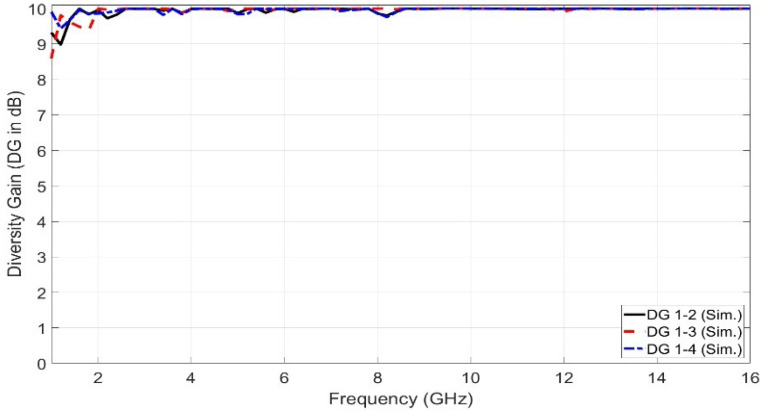
The simulated diversity gain versus frequency.

ECC represents the envelope correlation coefficient between the radiated antennas in the MIMO configuration. There are two methods of calculating the ECC. One is obtained from the S-parameter characteristics using Equation (6), while the other is obtained from the far-field pattern using Equation (7) [16]. ECC represents an important parameter that indicates the level of separation between the antennas in the MIMO structure. A low level of ECC indicates that there is a high level of separation. Ideally, the value of ECC should equal zero. Practically, the accepted limits of ECC are less than 0.5. The ECC based on the far-field results is more accurate than that based on the S-parameter as it includes the field coupling of the closely spaced antenna elements. Figure 24 shows the ECC curve generated from the far-field results. It is depicted from Figure 24 that the peak value of ECC generated from the far-field results does not exceed 0.05. Therefore, a very high degree of separation and isolation between the antennas in the proposed MIMO configuration is obtained. Moreover, there is an important parameter used in the UWB-MIMO antenna to measure the diversity performance, called the diversity gain (DG). Equation (8) was used to generate the DG curve. Figure 25 shows the diversity gain versus the operating frequency.

(6) $$ECC_{ij}=\frac{|S_{ii}^{*}S_{ij}+S_{ji}^{*}S_{jj}{|}^{2}}{\left(1-\right|S_{ii}{|}^{2}-|S{}_{j}i{|}^{2})(1-|S_{jj}{|}^{2}-|S_{ij}{|}^{2})}$$

where $S_{ii}$ and $S_{jj}$ are the reflection coefficients at port *i* and port *j*, respectively, while $S_{ij}$ and $S_{ji}$ are the isolation between port *i* and port *j*.

(7) $$ECC_{ij}=\frac{|\int_{0}^{2\pi }\int_{0}^{\pi }XPR\cdot E_{\theta i}\cdot E_{\theta j}^{*}\cdot P_{\theta }+E_{\phi j}\cdot E_{\phi j}^{*}\cdot P_{\phi }{)d\Omega |}^{2}}{\int_{0}^{2\pi }\int_{0}^{\pi }XPR\cdot E_{\theta i}\cdot E_{\theta i}^{*}\cdot P_{\theta }+E_{\phi i}\cdot E_{\phi i}^{*}\cdot P_{\phi })d\Omega \times \int_{0}^{2\pi }\int_{0}^{\pi }XPR\cdot E_{\theta j}\cdot E_{\theta j}^{*}\cdot P_{\theta }+E_{\phi j}\cdot E_{\phi j}^{*}\cdot P_{\phi })d\Omega }$$

where $ECC_{ij}$ is the envelope correlation coefficient between port *i* (i.e., antenna *i*) and port *j* (i.e., antenna *j*). The suffixes *i* and *j* represent the port numbers. In the case of *i* = 1 and *j* = 2, the calculated ECC is named ECC 1–2, which represents the ECC between port 1 and port 2. XPR is the cross-polarization ratio, and $P_{\theta }$ and $P_{\phi }$ are the angular density functions of the obtained wave in the θ-plane and ϕ-plane, respectively. $E_{\theta i}$ and $E_{\phi i}$ are the electric field components in the θ-plane and ϕ-plane for port *i*, respectively. $E_{\theta j}$ and $E_{\phi j}$ are the electric field components in the θ-plane and ϕ-plane for port *j*, respectively.

(8) $$DG=10\sqrt{1-{\left(ECC\right)}^{2}}$$

**Figure 26:**
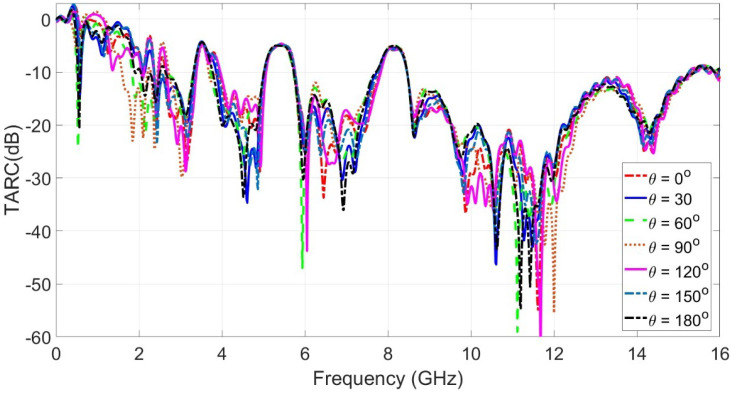
TARC curves at different values of theta from 0${}^{\circ }$ to 180${}^{\circ }$.

Another important parameter in terms of diversity characteristics is the total active reflection coefficient (TARC). The total active reflection coefficient (TARC) parameter shows the active operating spectrum for the proposed MIMO radiator configuration. Equation (9) is used to calculate the TARC parameter from the S-parameters.

(9) $$\Gamma_{a}^{t}=\sqrt{\frac{\left(\right|S_{11}+S_{12}e^{j\theta }{|}^{2})+(|S_{21}+S_{22}e^{j\theta }{|}^{2})}{2}}$$

where $S_{11}$ and $S_{22}$ are the reflection coefficients at port 1 and port 2, respectively. $S_{21}$ and $S_{12}$ are the transmission coefficients between port 1 and port 2. The θ is the phase value for the input signal. Figure 26 shows the TARC curves at different values of theta from 0${}^{\circ }$ to 180${}^{\circ }$.

Finally, Table 3 shows a comparison of the proposed UWB-MIMO antenna with other UWB-MIMO antennas.

## 5. Conclusions

Four ground-connected UWB-MIMO radiators were designed and fabricated on FR-4 dielectric substrate material with relative permittivity of $\varepsilon_{r}$ = 4.5, a loss tangent of 0.025, and a height of *h* = 1.5 mm. The UWB radiator element was proposed to operate in the FCC-UWB network. It has a bandwidth of 13.01 GHz extending from 2.03 to 15.04 GHz with triple band notches. The triple bands are the WiMAX band (3.28–3.8 GHz), WLAN band (5.05–5.9 GHz), and X-band (7.78–8.51 GHz). In addition to this, it supports the Bluetooth band (2.4–2.483 GHz). An alpha-shaped coupled line connected with a stub-loaded resonator (SLR) was proposed to achieve a band-stop characteristic at the WiMAX band. Two similar U-shaped slots etched on the radiator are utilized to obtain a band-stop characteristic WLAN band. Two unit cells of an octagon-shaped EBG structure are used to achieve a band-stop characteristic at X-band satellite communication. The three notch bands have excellent selectivity or sharpness at both the start and the end of each notch band. The UWB radiator is used in the MIMO configuration as an element. The ground planes of the four UWB radiators are connected together. Four DGS band-stop filters were placed between each ground plane and the center H-shaped ground connection to improve the matching characteristics at the Bluetooth band. In addition, high isolation characteristics were obtained at the operating band.

## Figures and Tables

**Table 1:** The optimum values for the proposed UWB antenna element design.

Parameter	Value (mm)	Parameter	Value (mm)	Parameter	Value (mm)	Parameter	Value (mm)
$L_{SUB}$	43	$L_{P7}$	1	$L_{C2}$	6.05	$L_{E2}$	3.24
$W_{SUB}$	30	$L_{P8}$	3.6	$L_{C3}$	2.35	$L_{E3}$	3.24
$W_{0}$	2.8	$L_{P9}$	2.5	$L_{C4}$	1.69	$L_{E4}$	1.62
$L_{G1}$	20.8	$L_{P10}$	2.5	$L_{C5}$	1.47	$L_{E5}$	1.35
$L_{G2}$	9.4	$L_{P11}$	3.4	$L_{C6}$	5.5	$L_{E6}$	2.36
$W_{G1}$	3.2	$L_{P12}$	3.4	$L_{C7}$	1	$L_{E7}$	1.86
$W_{G2}$	1.2	$L_{P13}$	3.4	$L_{C8}$	5.6	$W_{E1}$	0.27
$L_{P1}$	11.19	$L_{P14}$	1.08	$L_{C9}$	3.9	$W_{E2}$	0.25
$L_{P2}$	5.25	$W_{P1}$	12.84	$W_{C1}$	1.1	$W_{E3}$	0.2
$L_{P3}$	6	$W_{P2}$	1	$W_{C2}$	0.8	$D_{E1}$	1.2
$L_{P4}$	1.5	$W_{P3}$	0.75	$W_{C3}$	0.2	$D_{E2}$	0.3
$L_{P5}$	2.5	*R*	1	$W_{C4}$	0.2		
$L_{P6}$	1.5	$L_{C1}$	4.82	$L_{E1}$	1.12		

**Table 2:** The optimum values for the proposed UWB-MIMO antenna design.

Parameter	Value (mm)	Parameter	Value (mm)	Parameter	Value (mm)
$L_{G3}$	9.2	$L_{H1}$	8.5	$L_{D1}$	5
$L_{G4}$	5	$L_{H2}$	16	$L_{D2}$	6.1
$L_{G5}$	18	$W_{H1}$	1	$L_{D3}$	1.4
$L_{G6}$	13	$W_{H2}$	1	$L_{D4}$	1.4
$L_{G7}$	10.2	$W_{H3}$	0.5	$W_{D1}$	0.2
$L_{G8}$	15.5	$W_{H4}$	1.5	$W_{D2}$	0.2

**Table 3:** Performance comparison with existing literature.

Ref.	Size (mm${}^{3}$)	Dielectric Constant	Frequency Range (GHz)	Notch Bands (GHz)	Bluetooth Support	Isolation (dB)	Peak Gain (dBi)	Rad. Eff. (%)	ECC	Ground Planes(GP) Connection	Sharpness Edges at Notches
[18]	60 × 60 × 1.6	4.4	2.73–10.68	WLAN (5.36–6.04)	No	Below −15	5.5	N/A	<0.0015	No GP Connected	No
[19]	64 × 64 × 0.8	4.4	3–11	WLAN (5–6.1)	No	Below −40	5	80	<0.006	Connected	No
[20]	78 × 78 × 0.795	2.2	2.96–11.56	WLAN, WiMAX	No	Below −20	5.3	90	<0.107	No GP Connected	No
[21]	80 × 80 × 1.52	3	3.18–11.5	No Notches	No	Below −15	6	98	<0.012	No GP Connected	No
[22]	50 × 50 × 1.6	4.5	2–12	WLAN (4.85–6.35)	Yes	Below −17	5.8	86	<0.45	No GP Connected	No
[23]	60 × 60 × 1.24	4.4	3–11	No Notches	No	Below −20	3.4	75	<0.02	No GP Connected	No
[24]	80 × 80 × 1.6	4.4	2–20	WiMAX Radar App	Yes	Below −25	5.8	90	<0.05	No GP Connected	No
Proposed	78 × 78 × 1.5	4.5	2.33–16	WLAN, WiMAX, X-Band	Yes	Below −20	5.1	90	<0.05	Connected	Yes

## Data Availability

Not applicable.
